# A 6-year single-center prospective follow-up study of the efficacy of radiofrequency ablation for thyroid nodules

**DOI:** 10.3389/fendo.2024.1402380

**Published:** 2024-06-25

**Authors:** Shi Chuanke, Luo Ming, Yan Zhideng, Liu Huan

**Affiliations:** Department of General Surgery, Zhongshan Hospital of Traditional Chinese Medicine, Zhongshan, Guangdong, China

**Keywords:** radiofrequency ablation (RFA), volume reduction ratio (VRR), technique effective, regrowth, efficacy

## Abstract

**Background:**

Radiofrequency ablation (RFA) is an alternative modality for thyroid nodules (TNs) and many studies have also confirmed its favorable efficacy and safety. The scope of RFA increases in clinical practice and the aim of our study was to evaluate the efficacy of RFA.

**Methods:**

We conducted a prospective study to evaluate the efficacy of RFA for thyroid nodules between January 2017 and December 2022 at our institution. We assessed the change in nodal volume, volume reduction ratio (VRR), technique effective (TE) rate, complete ablation (CA) rate, and nodal regrowth rate and time after RFA.

**Results:**

We performed RFA for 1703 patients with TNs between January 2017 and December 2022, of which a total of 970 eligible patients were enrolled in the study. The preoperative volume of TNs was 6.23 ± 8.11ml, with 821 benign and 149 malignant nodules. The post-RFA TE and adjusted TE rate were 80% and 88.8%, respectively. CA was achieved in 145 (14.9%) patients with a mean time of 18.32± 12.98 months; nodal regrowth occurred in 15 (1.5%) patients with a mean time of 29.80 ± 12.47 months. TNs volume and VRR changed significantly at years 1 and 2 after RFA and stabilized after 5 years. A serious postoperative adverse event occurred in one patient with cervical sympathetic chain injury resulting in Horner’s syndrome. A transient or permanent damage of the recurrent laryngeal nerve could not be evaluated due to the lack of postoperative laryngoscopy, and this is a significant limitation of the study.

**Conclusion:**

The expanded RFA indications were also effective for TNs, with no significant change in long-term efficacy.

## 1 Introduction

Thyroid nodules (TNs) are very common in the general population up to 68% (1) and their incidence is also gradually increasing attributed to incidental nodule findings. It includes both benign and malignant nodules, however, benign nodules are more than 90%. Most of these benign nodules are clinically asymptomatic requiring only follow-up observation (2), and treatment is indicated for those with symptoms of compression (including important structures such as the trachea, recurrent laryngeal nerve, esophagus, etc.) or aesthetic concerns. More than 90% of malignant nodules are papillary thyroid carcinomas, which are indolent tumors. Surgery is the standard treatment for thyroid nodules; however, it leads to scar formation and, there are adverse events such as recurrent laryngeal nerve, hypothyroidism, and hypoparathyroidism after surgery (3, 4). Long-term hormone replacement therapy may be required for postoperative hypothyroidism, which may have adverse effects on the bone and cardiac system (5). Moreover, thyroid nodules are becoming more common among young people; therefore, more and more patients prefer minimally invasive treatments without surgical scars. Thermal ablation has emerged as an alternative minimally invasive treatment for thyroid nodules, including radiofrequency ablation (RFA), laser ablation (LA), microwave ablation (MA), and high-frequency focused ultrasound (HIFU).

RFA is the most commonly used thermal ablation technique, and many previous studies have confirmed that RFA has no significant difference in efficacy compared with surgery (6, 7). However, the risk of adverse events such as postoperative voice changes, hypothyroidism, and hypoparathyroid function was significantly lower (6–9). Currently, the indications for RFA in multiple guidelines are benign thyroid nodules, thyroid micropapillary carcinoma, recurrent lymph nodes in the neck, those who refuse thyroid surgery, or those who are ineligible for surgery due to systemic disease (5, 10, 11). However, due to the indolent characteristics of malignant nodules and aesthetic concerns, many studies have attempted to perform RFA for low-risk papillary thyroid carcinoma (12, 13). Moreover, the RFA threshold for benign nodules has not been clearly defined, and many studies have also applied RFA to larger nodules, like Deandrea M et al. for volumes >20 ml (14). Many previous studies have evaluated RFA separately for benign and malignant nodules (4, 11–14) and our study evaluated its efficacy for both. At the same time, these studies had small sample sizes, and we conducted a 6-year continuous prospective study with a larger sample size.

The aim of our study was to conduct a large sample size, consecutive prospective study to evaluate the efficacy of RFA for thyroid nodules, both benign and malignant.

## 2 Materials and methods

### 2.1 Patients

Our study was a single-center prospective study, which was approved by our Institutional Ethics Committee (No. 2022ZSY-LLK-456), and all subjects obtained informed consent prior to surgery. Patients with TNs who underwent RFA at Zhongshan Hospital of Traditional Chinese Medicine between January 2017 and December 2022 were enrolled. All thyroid nodules were evaluated for malignant risk by experienced sonographers based on the ACR TI-RADS grading system (15) prior to RFA. All patients also underwent fine-needle aspiration biopsy (FNA) of the TNs to clarify their nature prior to RFA.

The inclusion criteria for eligible patients were as follows: 1) RFA performed at our institution between January 2017 and December 2022; 2) TNs with TI-RADS grading; and 3) TNs with definitive FNA pathology results, including benign nodules and malignant nodules (papillary thyroid carcinoma). The exclusion criteria were as follows: 1) those who refused to participate in this study; 2) patients who were lost to follow-up; 3) incomplete data; and 4) those who received other RF treatments before RFA (LA, MA, HIFU).

### 2.2 RFA preoperative preparation and postoperative follow-up

All patients completed routine blood, biochemical tests, coagulation, thyroid function, electrocardiogram (ECG), and chest X-ray to exclude contraindications before RFA. The demographic characteristics of the patients included age, gender, and history of previous surgeries and diseases, especially thyroid surgery and treatment. All thyroid nodules were scored and risk stratified according to the ACR TI-RADS grading system.

After RFA, ultrasound(US) was performed at months 1, 3, 6, 12, and every 6 or 12 months thereafter. Thyroid function was assessed again 1 month after RFA. We followed up to assess whether there were events requiring emergency surgery or prolonged hospitalization after RFA.

### 2.3 RFA procedure

All RFAs were performed by experienced surgeons and sonographers in an outpatient setting applying a bipolar RFA generator and an 18-gauge bipolar radiofrequency electrode with a 0.9 cm active tip (CelonProSurge, Olympus Surgical Technologies, Germany). The patient was placed in the supine position with full neck extension, and local anesthesia with lidocaine was applied for pain control. The RFA was performed under real-time ultrasound guidance with hydro dissection, trans-isthmic approach, and the moving shot technique (16). Appropriate length electrodes were selected for the RFA procedure based on the size and location of the TNs, and contrast-enhanced ultrasonography (CEUS) was performed before and after RFA. During the RFA procedure, we assessed the patients for changes in voice, dyspnea, and other discomforts, and discharged them after 12 hours of postoperative observation without significant discomfort.

### 2.4 Variables

The variables in this study were general demographic characteristics and post-RFA efficacy indicators. Demographic characteristics included age, gender, underlying disease, thyroid-related disease and their treatment history. Post-RFA indicators included TNs volume, volume reduction ratio (VRR), technical effectiveness (TE), TNs regrowth, new onset, period of stabilization of TNs after RFA and re-intervention rate.

The three-dimensional size of the TNs was measured in ultrasound and its volume was calculated: Volume equation=[length(sagittal,cm)×depth(anteroposterior, cm)×width(transverse, cm)]×0.524. Volume Reduction Rate (VRR) is an important indicator for assessing the effectiveness of RFA and is calculated as follows:VRR = [(Initial Volume - Final Volume)×100%]/Initial Volume. Technical effectiveness (TE) was a >50% reduction in TNs volume at 12 months after RFA; TNs regeneration was defined as a 50% increase in total volume over the previous minimum volume (17).

### 2.5 statistical analysis

In our study, continuous variables were described by mean ± standard deviation (SD) and statistically analyzed by Students t test or Mann Whitney U test according to their distribution. Categorical variables are expressed in frequency (percentage), and statistical analysis is performed using Chi-square tests or Fisher exact tests when appropriate. We used multivariate logistic regression analysis to find the factors that affected the RFA effect, expressed by the adjusted Odds ratio (OR) and 95% confidence interval (CI). P < 0.05 was considered statistically significant. All data were analyzed using SPSS 25.0 version.

## 3 Results

**Figure 1:**
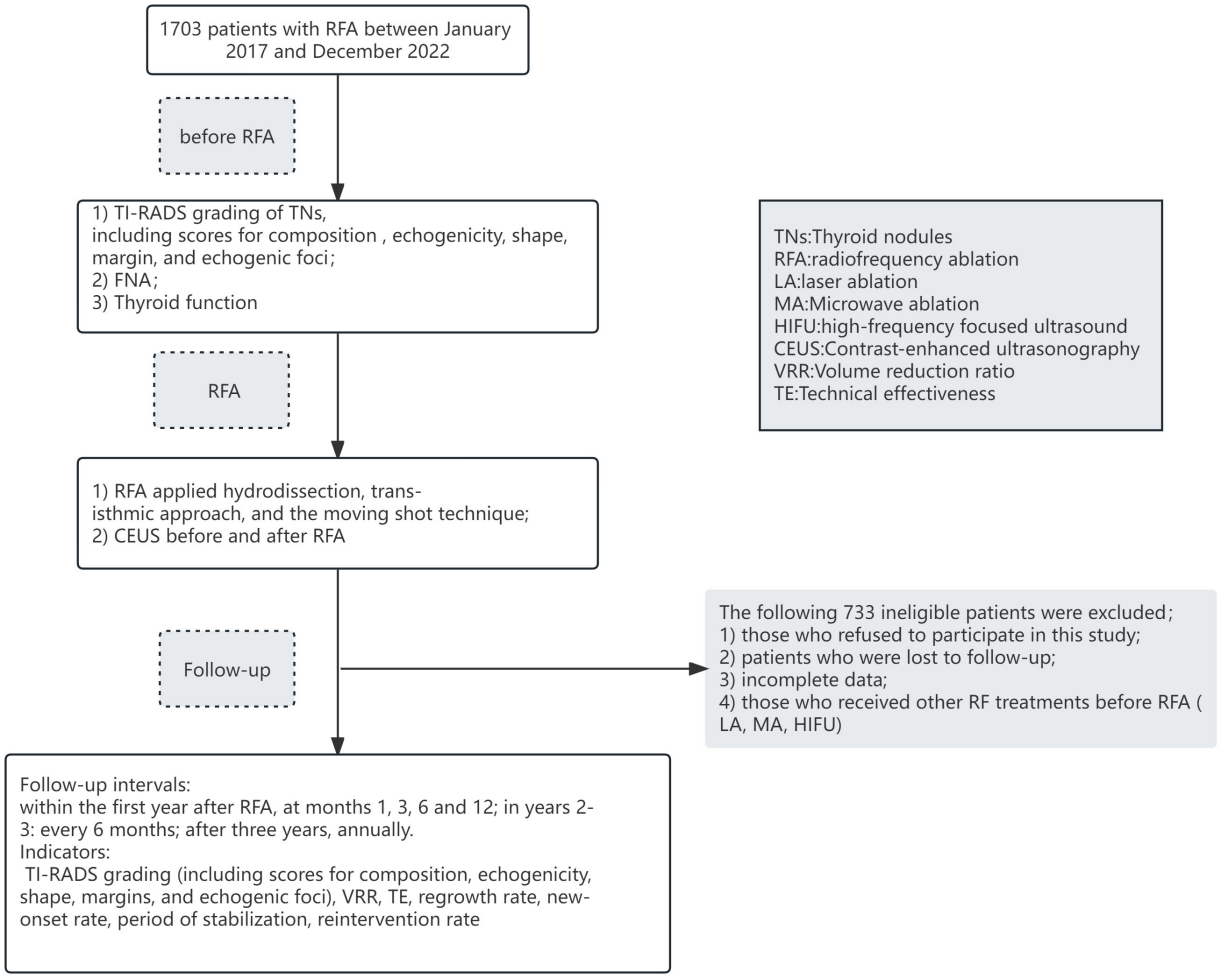
Flow chart of the study.

The flow of our study was shown in **Figure 1**. A total of 1703 patients with TNs underwent RFA between January 2017 and December 2022. After excluding 733 ineligible patients, a total of 970 eligible patients were included in our study. The demographic characteristics of all eligible patients are shown in **Table 1**. There were 803 females and 167 males, with minimum and maximum ages of 5 and 78 years, and a mean age of 43.63 ± 12.36 years. Of these patients, 654 (67.4%) had multiple TNs, and, 201 (20.7%) of those with multiple nodes underwent multiple node ablation treatments during a single RFA. 18 (1.9%) patients had previous RFA and 15 (1.5%) patients were postoperative nodules of residual glands for RFA. Preoperative FNA in RFA for TNs has confirmed 821 (84.6%) as benign and 149 (15.4%) as malignant.

**Table 1:** Clinical characteristics of the study.

Characteristic	n (%)
Genders	
Female/male n (%)	803/167(82.8%/17.2%)
Age, years,	
(min,max;mean ± SD)	(5,78; 43.63 ± 12.36)
Number of TNs	
1/>1 n (%)	(316/654) (32.6%/67.4%)
The number of TNs for RFA	
1/>1 n (%)	(769/201) (79.3%/20.7%)
History of prior RFA	
Yes/no n (%)	(952/18) (98.1%/1.9%)
Postoperative residual thyroid	
Yes/no n (%)	(15/955) (1.5%/98.5%)
Pathologic results of TNs	
Benign/Malignant n (%)	(821/149) (84.6%/15.4%)

SD, Standard deviation; TNs, Thyroid nodules; RFA, radiofrequency ablation.

The post-RFA follow-up and efficacy assessments were shown in **Table 2**. Of the 970 patients who were eligible for consecutive follow-up, the mean follow-up time was 17.60 ± 13.66 months, with the shortest and longest follow-up times being 1 month and 66 months, respectively. Technically effective (TE) was achieved in 776 patients after RFA, and their overall TE rate was 80%. However, because some patients were followed up for less than 12 months, their adjusted TE rate was 88.8% (776/874) after excluding the 96 unsuccessful TEs in these patients. Of these 970 patients, 145 patients achieved complete ablation (CA) with a CA rate of 14.9%, and the mean time to achieve CA was 18.32 ± 12.98 months. Regrowth of TNs occurred in 15 patients with a rate of 1.5% and its mean regrowth time was 29.80 ± 12.47 months.

**Table 2:** Follow up and efficacy of RFA.

Characteristic	n (%)
Patients (n)	970
Follow-up time, months	
(min,max)	(1,66)
(mean ± SD)	17.60 ± 13.66
TE rate n (%)	776/970, 80
TE* rate n (%)	776/874,88.8
CA rate, n (%)	145/970, 14.9
CA time, months	
(mean ± SD)	18.32 ± 12.98
Regrowth rate	15/970, 1.5
Regrowth time, months	
(mean ± SD)	29.80 ± 12.47

SD, Standard deviation; TE, Technical effectiveness; TE*, The adjusted technical effectiveness; CA, Complete ablation.

**Figure 2:**
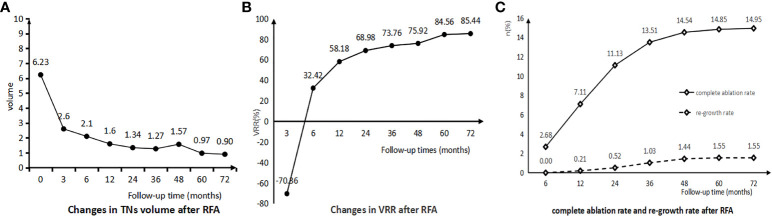
**(A)** Changes in TNs Volume after RFA. **(B)** Changes in TNs VRR after RFA. **(C)** Complete ablation rate and re-growth rate after RFA.

The changes in the volume of TNs after RFA were shown in **Table 3**; **Figures 2A, B**. The mean volume of TNs before RFA was 6.23 ± 8.11 ml. There was a gradual decrease in the volume of TNs after RFA, which was most significant at months 3 (2.60 ± 3.06 ml, p < 0.001 < 0.05) and months 12 (1.60 ± 2.30 ml, p = 0.004 < 0.05). The volume reduction rate (VRR) of TNs did not decrease at month 3 after RFA; on the contrary, an increase occurred (-70.36 ± 402.64%). The VRR gradually increased after months 3 and was most significant at postoperative 1 year (58.18 ± 76.86%, p < 0.001 < 0.05) and 2 years (68.98 ± 59.91%, p = 0.019 < 0.05). The complete ablation and re-growth rates of TNs after RFA were shown in **Table 4**; **Figure 2C**. The CR rates were 7.11%, 11.13%, 13.51%, 14.54%, 14.85%, and 14.95% at 1, 2, 3, 4, 5, and 6 years after RFA, respectively. Re-growth rates at 1, 2, 3, 4, 5, and 6 years after RFA were 0.21%, 0.52%, 1.03%, 1.44%, 1.55%, and 1.55%, respectively.

**Table 3:** Volume and VRR of TNs.

	Volume (mean ± SD,ml)	P-value	VRR (mean± SD,%)	P-value
preoperative	6.23 ± 8.11		–	–
Post-operative			–	–
1–3 months	2.60 ± 3.06	<0.001	-70.36 ± 402.64	–
3–6 months	2.10 ± 3.01	0.297	32.42 ± 137.36	<0.001
6–12 months	1.60 ± 2.30	0.004	58.18 ± 76.86	<0.001
12–24months	1.34 ± 2.08	0.077	68.98 ± 59.91	0.019
24–36months	1.27 ± 2.00	0.670	73.76 ± 33.58	0.321
36–48months	1.57 ± 2.39	0.259	75.92 ± 33.60	0.607
48–60months	0.97 ± 1.52	0.089	84.56 ± 19.34	0.151
>60months	0.90 ± 0.97	0.930	85.44 ± 15.59	0.601

TNs, Thyroid nodules; VRR, Volume reduction rate; SD, Standard deviation.

**Table 4:** Complete ablation rate and re-growth rate after RFA.

	Complete ablation rate n(%)	P-value	Regrowth rate n(%)	P-value
Post-operative				
0–6 months	6(2.68)	–	0(0)	–
6–12 months	69(7.11)	<0.001	2(0.21)	0.949
12–24months	108(11.13)	<0.001	5(0.52)	0.452
24–36months	131(13.51)	0.128	10(1.03)	0.300
36–48months	141(14.54)	0.556	14(1.44)	0.539
48–60months	144(14.85)	0.898	15(1.55)	0.999
>60months	145(14.95)	0.949	15(1.55)	0.999

RFA, radiofrequency ablation.

## 4 Discussion

Thyroid nodules (TNs) are extremely common in the general population, and their incidence is increasing due to the popularity of high-resolution ultrasound and the emphasis on health issues. Many previous studies have confirmed that RFA is an optional safe and effective treatment for TNs. The majority of TNs are benign nodules and more than 90% of malignant nodules are papillary thyroid carcinomas with a biological predisposition to indolence; similarly, patients have aesthetic concerns, all of which contribute to a higher incidence of RFA. As a result, the scope of RFA has gradually expanded from benign nodules to micropapillary thyroid cancer and even to recurrent thyroid cancer and low-risk papillary thyroid cancer.

Surgery is still the mainstay of treatment for TNs, and its postoperative risk of adverse events such as hypothyroidism, hypoparathyroidism, and recurrent laryngeal nerve injury is significantly higher than that of RFA (4, 18, 19). Surgery involves removal of the thyroid gland with nodules, whereas RFA involves coagulative necrosis of the nodules into biologically inactive scar tissue by thermal ablation; therefore, TNs do not immediately decrease or disappear in apparent physical size after RFA, and may even show enlargement in the short-term. TNs volume and volume reduction rate (VRR) are important indicators for assessing the efficacy of RFA. In our study, the volume of TNs decreased from 6.23 ml preoperatively to 1.6 ml, 1.34 ml, 1.27 ml, 1.57 ml, 0.97 ml, and 0.90 ml at postoperative 1, 2, 3, 4, 5, and 6 years, respectively and its VRR was 58.18%, 68.98%, 73.76%, 75.92%, 84.56% and 85.44% at postoperative 1, 2, 3, 4, 5 and 6 years, respectively. Volume reductions in TNs were most significant at years 1 and 2 after RFA, and it stabilized after 2 years. A meta-analysis showed that benign TNs had a VRR of 75% and 87% at years 1 and 2 after RFA, respectively (4). In another meta-analysis including 24 studies of ablation of benign TNs showed a VRR of 66% and 62% at years 1 and 2 after RFA, respectively; these are consistent with our study (20). Kim MK et al. found that the VRR of malignant nodules was lower than that of benign ones at 12 months after RFA (51.4% versus 83.8%, P = 0.01 < 0.05) (21). A study of 74 patients with thyroid micropapillary carcinoma (PTMC) followed for more than 5 years found that RFA resulted in complete disappearance of the tumor lesions without local tumor progression, lymph node or distant metastasis (22). In our study, we saw complete ablation of TNs lesions in 145 (4.9%) patients, which occurred with a mean follow-up time of 18.32 ± 12.98 months.

RFA treatment is not the removal of glands with clinically significant nodules; therefore, patients are more anxious and concerned about the recurrence of nodules after RFA. Many studies have demonstrated that incomplete ablation resulting in residual nodules at the margins is an important factor in regeneration (23, 24). It has been also noted that incomplete ablation of benign nodules may promote nodule growth to some extent (25). However, many studies indicated that the initial ablation ratio (IAR) did not correlate with nodal regrowth, but rather with VRR and the likelihood of retreatment (25–28). Performing CEUS before and after RFA for more complete ablation reduces the possibility of residual nodules; however, factors such as large nodules, restricted anatomical locations V(29), and large calcified foci (30) affect ablation efficacy, and identification of accurate borders leads to residual nodules after initial ablation. Although previous studies have indicated that the primary purpose of RFA for benign nodules is to alleviate compression symptoms rather than complete ablation (31), many studies have confirmed its favorable safety and efficacy, allowing it to treat PTMC, recurrent thyroid cancer, and even low-risk papillary thyroid cancer. Therefore, the current aims of RFA are not only to improve symptoms, but also to eliminate tumor lesions (12, 13, 22). Those puncture-proven benign nodules that do not achieve satisfactory regression or regeneration after RFA may actually be malignant. Many studies have also confirmed its and found that regenerating nodules after RFA were confirmed to be malignant during subsequent surgeries (32, 33). Therefore, for these nodules that do not significantly regress or regenerate after RFA, a puncture biopsy is recommended to exclude the possibility of malignancy and guide subsequent treatment.

In our study, we saw a significant reduction in the volume of TNs after RFA at 1 and 2 years, and its stabilization at 2 years. A meta-analysis also showed that the volume of benign nodules decreased rapidly within 12 months after ablation, with a plateau at months 12 - 36 (34). The change in VRR was consistent with the change in nodal volume, which also stabilized at 2 years. The VRR showed a negative increase in 3 months after RFA, which is consistent with previous studies; it is due to the ablation area exceeding the boundaries of the nodule (especially small nodules), and changes such as peripheral edema and inflammation affecting the definition of the boundaries in the early post-RFA period. Regrowth of the nodules predominantly started in the 2nd year after RFA and stabilized in the 4th year. Previous studies have also confirmed that regrowth occurs after 2 and 3 years (35–37). However, Sim JS et al. found that its regrowth shows a peak during the 2nd-3rd year after RFA and another peak after the 5th year (23). Valcavi R et al. found that regrowth is rare after the 4th year (38), which is consistent with our findings. These differences may be due to inconsistencies in follow-up times, leading to different definitions of minimum volume; therefore, Mauri, G et al. suggested that regrowth be defined as a 50% increase in the minimum recorded volume compared to that measured at a given follow-up time point (17).

Parameters regarding the prediction of regrowth after RFA have not been clarified (37). Yan et al. found that residual active nodule rate, initial volume, location, and vascular distribution were all independent risk factors associated with regrowth (28). Negro R et al. found that VRR at 12 months after RFA was associated with regrowth (35). An increase in the volume of the residual active nodule may be an early sign of nodule regrowth (27, 39). Many studies also indicated that margin re-expansion is an important cause of recurrence after RFA (23, 29, 40). There is currently no consensus on the timing and indications for reintervention in these regrowing nodules. Some studies have indicated that a single treatment for benign nodules treated to relieve compression symptoms or improve cosmetic problems is sufficient, even if nodule regrowth occurs (31). Kim HJ et al. demonstrated that single RFA is effective for small nodules without initial regrowth or symptomatic recurrence. However, additional treatments improved the VRR for nodules with regrowth, increased Vv, or symptomatic recurrence (41). One study found that a VRR of <66% at 1 year after RFA was a better predictor of nodal retreatment, whereas young age and large initial volume may also be associated with its retreatment (37). Another study found that an IAR >73% was a good predictor of no retreatment within 5 years after RFA (26). The study showed that the energy delivered during RFA is also a reliable predictor of retreatment (37). Therefore, avoiding residual active nodules after RFA reduces the likelihood of nodule regrowth or retreatment. Preoperative CEUS identified the targeting area and postoperative CEUS confirmed complete ablation of the targeting area. Additional RFA is indicated for patients with larger nodules, unresolved clinical problems, or regrowth or increased Vv after initial ablation. Some studies have also confirmed that subsequent RFA for large benign nodules improves VRR and efficacy. However, the optimal timing and indications for reintervention, including RFA and invasive procedures, need to be further explored.

Our study has its own limitations as follows: 1. We followed up only for major complications of E and F classifications as defined by the Society of Interventional Radiology (SIR) (42). A serious postoperative adverse event occurred in one patient with cervical sympathetic chain injury resulting in Horner’s syndrome. A transient or permanent damage of the recurrent laryngeal nerve could not be evaluated due to the lack of postoperative laryngoscopy, and this is a significant limitation of the study. 2. Most thyroids are multiple nodules, and many patients had multiple nodules ablated during a single RFA; however, we only evaluated large nodules. Therefore, whether there is a difference between ablation of multiple nodules versus single nodules during the same RFA, and whether ablation has an effect on untreated nodules (both ipsilateral and contralateral); none of the above were involved in this study and will be explored in our subsequent studies.

## Data availability statement

The original contributions presented in the study are included in the article/supplementary material. Further inquiries can be directed to the corresponding author.

## Author contributions

SC: Supervision, Writing – review & editing, Project administration, Methodology, Investigation, Data curation, Conceptualization. LM: Writing – review & editing, Supervision, Methodology. YZ: Investigation, Writing – review & editing, Supervision, Methodology. LH: Writing – original draft, Project administration, Formal Analysis, Data curation, Conceptualization, Writing – review & editing, Methodology, Investigation.
